# Prognostic and Immunological Role of FUN14 Domain Containing 1 in Pan-Cancer: Friend or Foe?

**DOI:** 10.3389/fonc.2019.01502

**Published:** 2020-01-10

**Authors:** Qingchen Yuan, Na Sun, Jiayu Zheng, Yingxuan Wang, Xiaole Yan, Wuqian Mai, Yuhua Liao, Xiao Chen

**Affiliations:** ^1^Department of Cardiology, Tongji Medical College, Union Hospital, Huazhong University of Science and Technology, Wuhan, China; ^2^Key Lab of Molecular Biological Targeted Therapies of the Ministry of Education, Tongji Medical College, Union Hospital, Huazhong University of Science and Technology, Wuhan, China; ^3^Department of Pathogen Biology, School of Basic Medicine, Tongji Medical College, Huazhong University of Science and Technology, Wuhan, China

**Keywords:** mitophagy, pan-cancer, database, survival analysis, immune infiltration, tumor microenvironment

## Abstract

**Background:** FUN14 domain containing 1 (FUNDC1) plays a pivotal role in mitochondrial autophagy (mitophagy), which is closely associated with human immunity. However, the role of FUNDC1 in cancers remains unclear. This study aimed to visualize the prognostic landscape of FUNDC1 in pan-cancer and investigate the relationship between FUNDC1 expression and immune infiltration.

**Methods:** In this study, we explored the expression pattern and prognostic value of FUNDC1 in pan-cancer across multiple databases, including ONCOMINE, PrognoScan, GEPIA, and Kaplan-Meier Plotter. Then, using the GEPIA and TIMER databases, we investigated the correlations between FUNDC1 expression and immune infiltration in cancers, especially liver hepatocellular carcinoma (LIHC), and lung squamous cell carcinoma (LUSC).

**Results:** In general, compared with that in normal tissue, tumor tissue had a higher expression level of FUNDC1. Although FUNDC1 showed a protective effect on pan-cancer, a high expression level of FUNDC1 was detrimental to the survival of LIHC patients. Although different from what was found for LUSC, for LIHC, there were significant positive correlations between FUNDC1 expression and immune infiltrates, including B cells, CD8+ T cells, CD4+ T cells, neutrophils, macrophages, and dendritic cells. Furthermore, markers of infiltrating immune cells, such as tumor-associated-macrophages (TAMs), exhibited different FUNDC1-related immune infiltration patterns.

**Conclusion:** The mitophagy regulator FUNDC1 can serve as a prognostic biomarker in pan-cancer and is correlated with immune infiltrates.

## Introduction

As the power plants in the human cells mitochondria generate adenosine triphosphate by oxidative phosphorylation to fuel cellular activities while also producing reactive oxygen species, which damage them. To maintain a proper balance, mitochondria undergo fission-fusion cycles and eliminate damaged and redundant sections by mitochondrial autophagy (mitophagy), a process that requires various molecular interactions during mitochondrial quality control (1–3).

FUN14 domain containing 1 (FUNDC1), which anchors to the outer mitochondrial membrane, is pivotal in mitophagy (4, 5). FUNDC1 interacts with molecules like LC3B, which is found on the mitochondria-associated membrane of the endoplasmic reticulum, to maintain good mitochondrial quality by forming mitophagosomes (4). FUNDC1-related mitochondrial dysfunction contributes to various pathophysiological processes, such as heart diseases, metabolic disorders, and cancers (6–9). In cardiovascular and metabolic diseases, FUNDC1 is generally considered to be protective because FUNDC1-mediated mitophagy can alleviate damage caused by intracellular stress such as hypoxia and thus benefit overall outcomes (9, 10). However, unlike in non-cancerous diseases, the role of FUNDC1 in pan-cancer has been largely underexplored. Meanwhile, the role of FUNDC1 might be context dependent and could vary among different cancers. For example, FUNDC1 can promote tumor progression and predict poor prognosis in some cancer types; however, it can also suppress carcinogenesis through mitophagy (6, 7, 11). Thus, it remains unclear whether FUNDC1 can be characterized as a friend or foe in pan-cancer.

The tumor microenvironment (TME) contains various cells. Among them, infiltrating immune cells account for a large proportion (12). On the one hand, unlike the conventional view of immune cells as a component of an antitumor strategy, immune infiltration into the TME reflects a tactic tumor cells use to avoid being killed (13–15). For example, tumor-associated-macrophages (TAMs) can help tumor cells in several ways, including immune escape, tumor angiogenesis, and metastasis (16–19). In addition, aside from macrophages, almost all types of immune cells, including B cells, CD8+ T cells, CD4+ T cells, neutrophils, natural killer (NK) cells, and dendritic cells (DC), are found in the TME, and some participate in the development of cancers (12). In contrast, immunotherapy targeting interactions between immune cells and tumor cells, as an alternative approach to classic anticancer treatments, have been developed in recent years to reactivate adaptive and innate immune systems and create a robust antitumoral immune response. For example, cytotoxic T lymphocyte associated antigen 4 (CTLA4), programmed death-1 (PD-1), and programmed death ligand-1 (PD-L1) inhibitors were found to have promising antitumor effects on malignant melanoma and non-small-cell lung carcinoma (14, 20). However, only a limited proportion of patients with certain cancer types respond well to current immunotherapies (14). Thus, it is necessary to explore additional potential targets.

In this study, we visualized the prognostic landscape of FUNDC1 in pan-cancer using databases, including ONCOMINE, PrognoScan, GEPIA, and Kaplan-Meier Plotter. We then explored the potential relationships between FUNDC1 expression and immune infiltration levels using the TIMER and GEPIA databases. The findings from this study indicate that FUNDC1 influences the prognosis of patients with cancers, probably via its interaction with infiltrating immune cells.

## Materials and Methods

### FUNDC1 Expression in Human Cancers in ONCOMINE

The mRNA expression of FUNDC1 in different cancer types was analyzed in the ONCOMINE database (www.oncomine.org). The thresholds were set as a *P*-value of 0.001 and fold change of 1.5.

### Survival Analysis in PrognoScan, GEPIA, and Kaplan-Meier Plotter

The correlation between FUNDC1 expression and survival in pan-cancer was analyzed in PrognoScan (http://dna00.bio.kyutech.ac.jp/PrognoScan/index.html), Kaplan-Meier Plotter (https://kmplot.com/analysis/), and GEPIA (http://gepia.cancer-pku.cn/) (21–23). Specifically, the FUNDC1 expression level was searched in all available microarray datasets of PrognoScan to determine its relationship with prognosis, including overall survival (OS) and disease-free survival (DFS). The threshold was set as a Cox *P*-value < 0.05, and R software (version 3.25.0, www.r-project.org) with the “forestplot” package was utilized to summarize and visualize the survival analysis from PrognoScan. GEPIA is an interactive online platform with tumor sample information from TCGA and normal sample information from the TCGA and GTEx projects. We explored the effect of FUNDC1 expression on OS and DFS in each available cancer type (total number = 34). Kaplan-Meier Plotter is a powerful online tool that can be used to assess the effect of 54,000 genes on survival in 21 cancer types. We analyzed the relationship of FUNDC1 expression with overall survival (OS) and relapse-free survival (RFS) in liver hepatocellular carcinoma (LIHC), lung squamous cell carcinoma (LUSC), bladder carcinoma (BC), head and neck squamous cell carcinoma (HNSC), ovarian carcinoma (OV), breast invasive carcinoma (BRCA), lung adenocarcinoma (LUAD), and rectum adenocarcinoma (READ). Hazard ratios (HRs) with 95% confidence intervals (CI) and log-rank *P*-values were calculated.

### Correlations Between FUNDC1 Expression and Immune Cells in TIMER and GEPIA

The relationship between FUNDC1 expression and immune infiltration was determined using the TIMER (http://cistrome.org/TIMER/) and GEPIA databases (23, 24). TIMER is an ideal resource for the systematic analysis of immune infiltration across diverse cancer types. TIMER applies a previously published statistical deconvolution method to infer the abundance of tumor-infiltrating immune cells from gene expression profiles (24). The TIMER database contains 10,897 samples across 32 cancer types from TCGA to allow the evaluation of the abundance of immune infiltration. We analyzed FUNDC1 expression with the abundance of all six types of immune infiltrating cells, including B cells, CD4+ T cells, CD8+ T cells, neutrophils, macrophages, and dendritic cells. The relationship between the expression level of FUNDC1 and tumor purity was also determined.

In addition to the general analysis of immune cell type, we also analyzed the correlation between FUNDC1 expression and several immune cell markers to identify the potential subtypes of infiltrating immune cells. Immune gene markers were selected from the website of R&D Systems (https://www.rndsystems.com/cn/resources/cell-markers/immune-cells). These gene markers include markers of B cells, CD8+ T cells, follicular helper T cells (Tfh), T-helper 1 (Th1) cells, T-helper 2 (Th2) cells, T-helper 9 (Th9) cells, T-helper 17 (Th17) cells, T-helper 22 (Th22) cells, Tregs, exhausted T cells, M1 macrophages, M2 macrophages, tumor-associated macrophages, monocytes, natural killer (NK) cells, neutrophils, and dendritic cells. The gene expression level was adjusted with log_2_ RSEM. FUNDC1 was plotted on the x-axis, while marker genes were plotted on the y-axis. Scatterplots were used to analyze correlations between FUNDC1 and each immune gene marker. Similarly, in GEPIA, gene expression correlation analysis was performed for given sets of TCGA expression data. The Spearman method was used to determine the correlation coefficient. FUNDC1 was plotted on the x-axis, while other genes of interest were plotted on the y-axis.

### Statistical Analysis

The results generated in Oncomine are presented with P-values determined in *t*-tests, fold changes, and gene ranks. The Kaplan-Meier method was used to estimate the survival curve. To compare survival curves, we used the log rank test to calculate the HR and logrank *P*-value in Kaplan-Meier Plotter and GEPIA. A univariate Cox regression model was used to calculate the HR and Cox P value in PrognoScan. The correlation of gene expression was evaluated using Spearman's correlation. A *P* < 0.05 was considered statistically significant, if not specially noted.

## Results

### mRNA Expression Level of FUNDC1 in Pan-Cancer

**Figure 1:**
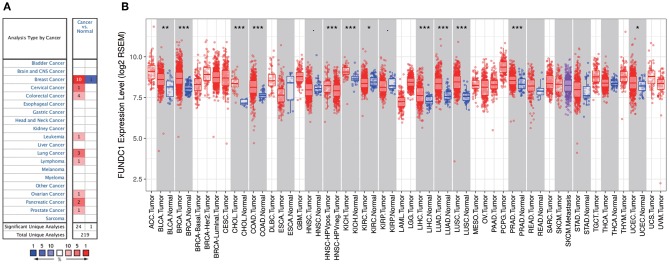
FUNDC1 expression levels in cancers. **(A)** Increased or decreased expression of FUNDC1 in different cancer tissues, compared with normal tissues in ONCOMINE. Number in each cell is the amount of datasets. **(B)** Human FUNDC1 expression levels in different cancer types from TCGA data in TIMER. *P < 0.05, **P < 0.01, ***P < 0.001.

FUNDC1 mRNA expression levels were analyzed in Oncomine to examine FUNDC1 expression over a cancer-wide range. The results revealed that compared with that in the respective normal groups, FUNDC1 expression was higher in cancer groups, including breast, cervical, colorectal, lung, ovarian, pancreatic, and prostate cancers as well as leukemia and lymphoma. Meanwhile, a lower expression of FUNDC1 was only found in one breast cancer dataset (Figure 1A). The details of FUNDC1 expression in multiple cancers are summarized in Supplementary Table 1.

To further evaluate FUNDC1 expression in pan-cancer, we examined RNA sequencing data in TCGA using TIMER. The differential FUNDC1 expression patterns in tumor and adjacent normal tissues are shown in Figure 1B. FUNDC1 expression was significantly lower in KIRC (kidney renal clear cell carcinoma) than in normal tissue. Meanwhile, FUNDC1 expression was significantly higher in BLCA (bladder urothelial carcinoma), BRCA (breast invasive carcinoma), CHOL (cholangiocarcinoma), COAD (colon adenocarcinoma), HNSC-HPV positive (head and neck squamous carcinoma-HPV positive), KICH (kidney chromophobe), LIHC (liver hepatocellular carcinoma), LUAD (lung adenocarcinoma), LUSC (lung squamous cell carcinoma), PRAD (prostate adenocarcinoma), and UCEC (uterine corpus endometrial carcinoma) than in their respective adjacent normal tissues.

### Multifaceted Prognostic Value of FUNDC1 in Cancers

**Figure 2:**
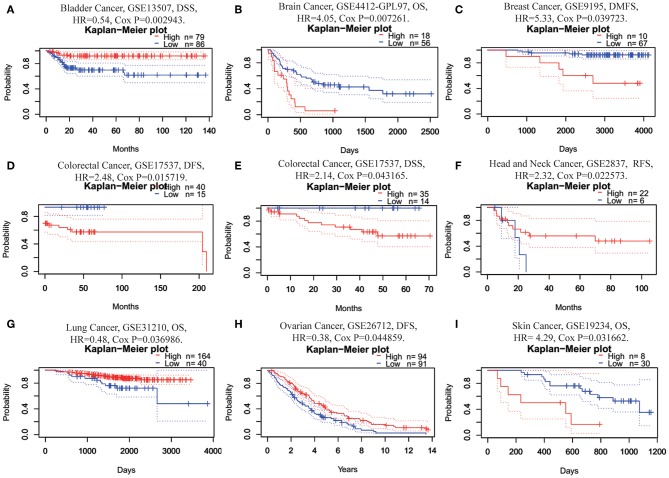
Kaplan-Meier survival curves comparing high and low expression of FUNDC1 in different cancer types in PrognoScan. **(A)** DSS (*n* = 165) in bladder cancer cohort GSE13507. **(B)** OS (*n* = 74) in brain cancer cohort GSE4412-GPL97. **(C)** DMFS (*n* = 77) in breast cancer cohort GSE9195. **(D,E)** DFS (*n* = 55) and DSS (*n* = 49) in colorectal cancer cohort GSE17537. **(F)** RFS (*n* = 28) in head and neck cancer cohort GSE2837. **(G)** OS (*n* = 204) in lung cancer cohort GSE31210. **(H)** DFS (*n* = 185) in ovarian cancer cohort GSE26712. **(I)** OS (*n* = 38) in skin cancer cohort GSE19234. DSS, disease-specific survival; OS, overall survival; DMFS, distant metastasis-free survival; DFS, disease-free survival; RFS, relapse-free survival.

Next, we investigated the prognostic value of FUNDC1 for pan-cancer in different databases. In PrognoScan, we explored the relationships between FUNDC1 expression and the prognosis of each cancer. The results are summarized in Supplementary Figure 1. Notably, FUNDC1 expression was significantly correlated with a total of eight cancer types, including bladder, brain, breast, colorectal, head and neck, lung, ovarian, and skin cancers (Figure 2). Among them, FUNDC1 played a detrimental role in five cancer types, including brain (OS: total number = 74, HR = 4.05, Cox *P* = 0.007261), breast [DMFS (distant metastasis-free survival): total number = 77, HR = 5.33, Cox *P* = 0.039723], colorectal [DFS (disease-free survival): total number = 55, HR = 2.48, Cox *P* = 0.015719; DSS (disease-specific survival): total number = 49, HR = 2.14, Cox *P* = 0.043165], head and neck [RFS (relapse-free survival): total number = 28, HR = 2.32, Cox *P* = 0.022573], and skin cancers (OS: total number = 38, HR = 4.29, Cox *P* = 0.031662). Meanwhile, FUNDC1 had a protective role in the other 3 cancer types, including bladder (DSS: total number = 165, HR = 0.54, Cox *P* = 0.002943), lung (OS: total number = 204, HR = 0.48, Cox *P* = 0.036986), and ovarian cancers (DFS: total number = 185, HR = 0.38, Cox *P* = 0.044859).

**Figure 3:**
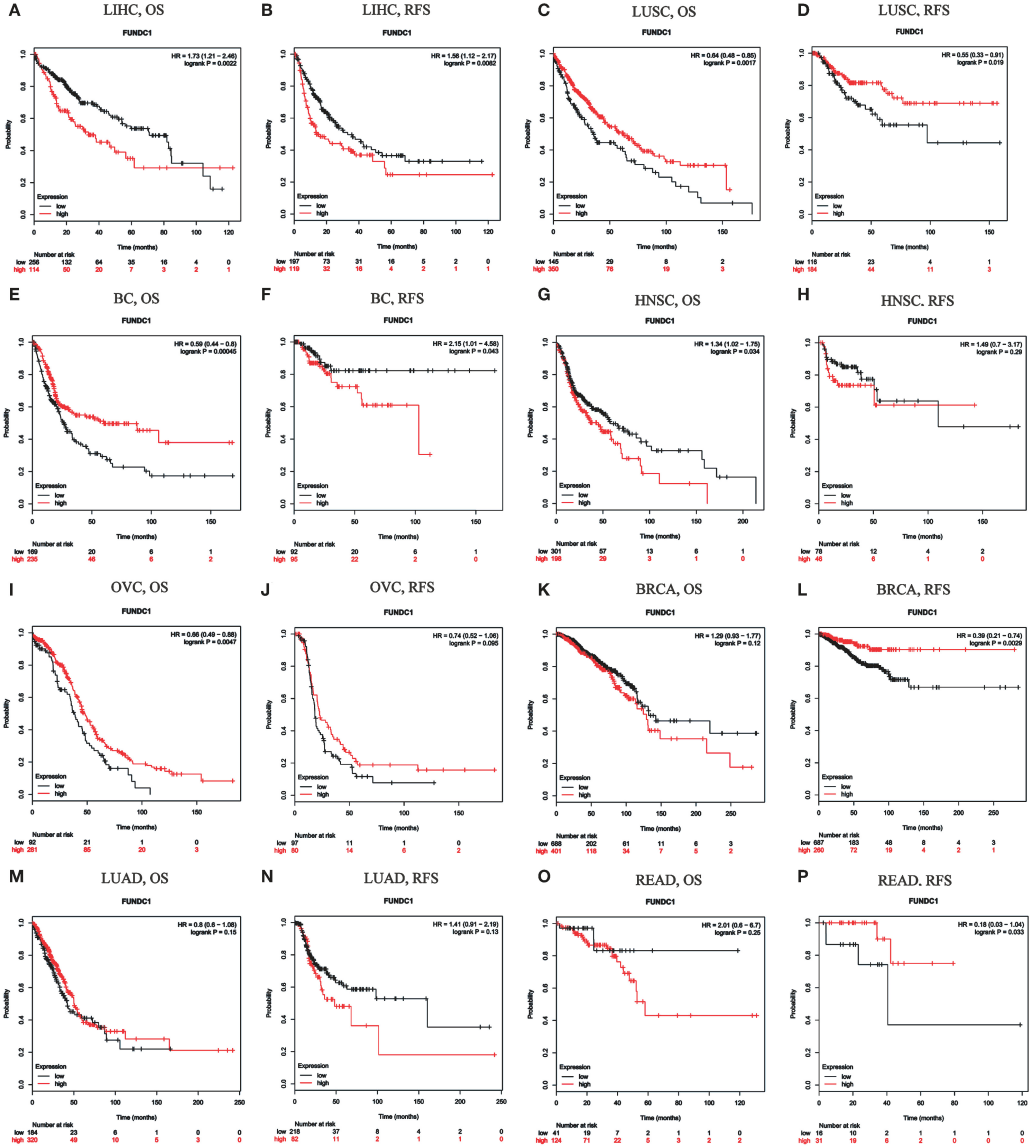
Kaplan-Meier survival curves comparing the high and low expression of FUNDC1 in different types of cancer in Kaplan-Meier Plotter. OS and RFS of **(A,B)** liver hepatocellular carcinoma (LIHC) **(C,D)** lung squamous cell carcinoma (LUSC) **(E,F)** bladder cancer (BC) **(G,H)** head and neck squamous cell carcinoma (HNSC) **(I,J)** ovarian cancer (OVC) **(K,L)** breast carcinoma (BRCA) **(M,N)** lung adenocarcinoma (LUAD), and **(O,P)** rectum adenocarcinoma (READ). Red curve represents patients with high expression of FUNDC1. OS, overall survival; RFS, relapse-free survival.

Using Kaplan-Meier Plotter, which is mainly based on Affymetrix microarray information from TCGA, we further assessed FUNDC1-related survival (OS and RFS) because the data in PrognoScan are mainly extracted from the gene expression omnibus (GEO) database. Interestingly, we newly identified FUNCD1 as a detrimental prognostic factor in LIHC (OS: HR = 1.73, 95% CI from 1.21 to 2.46, logrank *P* = 0.0022; RFS, HR = 1.56, 95%CI from 1.12 to 2.17, logrank *P* = 0.0082) (Figures 3A,B). This finding may challenge the previously reported protective role of FUNDC1 in hepatocellular carcinogenesis (11). The findings for lung cancer were partly different from those using PrognoScan, as a high expression of FUNDC1 only benefited LUSC (OS: HR = 0.64, 95 % CI from 0.48 to 0.85 logrank *P* = 0.0017; RFS: HR = 0.55, 95% CI from 0.33 to 0.91, logrank *P* = 0.019) (Figures 2C,D) and not LUAD (OS: HR = 0.8, 95% CI from 0.6 to 1.08, logrank *P* = 0.15; RFS: HR = 1.41, 95% CI from 0.91 to 2.19, logrank *P* = 0.13) (Figures 3M,N). For bladder cancer, FUNDC1 was found to have a protective effect on overall survival (OS: HR = 0.59, 95% CI from 0.44 to 0.8, logrank *P* = 0.00045). However, FUNDC1 worsened relapse-free survival in bladder cancer (RFS: HR = 2.15, 95% CI from 1.01 to 4.58, logrank *P* = 0.043) (Figures 3E,F). For both head and neck squamous cell carcinoma (HNSC) and ovarian cancer (OV), FUNDC1 significantly influenced their overall survival (HNSC: OS, HR = 1.34, 95% CI from 1.02 to 1.75, logrank *P* = 0.034; OV: OS, HR = 0.66, 95% CI from 0.49 to 0.88, logrank *P* = 0.0047) but not relapse-free survival (HNSC: RFS, HR = 1.49, 95% CI from 0.7 to 3.17, logrank *P* = 0.29; OVC: RFS, HR = 0.74, 95% CI from 0.52 to 1.06, logrank *P* = 0.095) (Figures 3G–J). For BRCA, FUNDC1 had a protective effect on relapse-free survival (RFS: HR = 0.39, 95% CI from 0.21 to 0.74, logrank *P* = 0.0029) but did not have a significant effect on overall survival (OS: HR = 1.29, 95% CI from 0.93 to 1.77, logrank *P* = 0.12) (Figures 3K,L). In addition, for colorectal cancer, FUNDC1 only had a protective effect on RFS for rectum adenocarcinoma (READ) (OS: HR = 2.01, 95% CI from 0.6 to 6.7, logrank *P* = 0.25; RFS: HR = 0.18, 95% CI from 0.03 to 1.04, logrank *P* = 0.033) (Figures 3O,P).

In addition to the microarray analysis of FUNDC1 in PrognoScan and Kaplan-Meier Plotter, we also utilized GEPIA to analyze RNA sequencing data in TCGA. In GEPIA, we analyzed the role of FUNDC1 in each cancer type (number of cancer types = 33), as well as the overall effect of FUNDC1 on cancers. In general, FUNDC1 was a favorable prognostic marker in cancers (OS: total number = 9,496, HR = 0.72, logrank *P* = 0; DFS: total number = 9,496, HR = 0.8, logrank *P* = 6.6E−09) (Supplementary Figure 2A). Specifically, compared with a low expression level, a high expression level of FUNDC1 was correlated with a better OS in KIRC and LUSC and DFS in thyroid carcinoma (THCA). On the contrary, compared with a low expression level, a high expression level of FUNDC1 was correlated with a poorer OS in brain lower grade glioma (LGG) and LIHC. In addition, unlike the findings from PrognoScan and Kaplan-Meier Plotter, FUNDC1 expression impacted neither OS nor DFS in BRCA, CHOL, COAD, HNSC, KICH, LUAD, OV, READ, and UCEC (Supplementary Figure 2).

### FUNDC1 Expression in a Stratified LIHC Population

**Figure 4:**
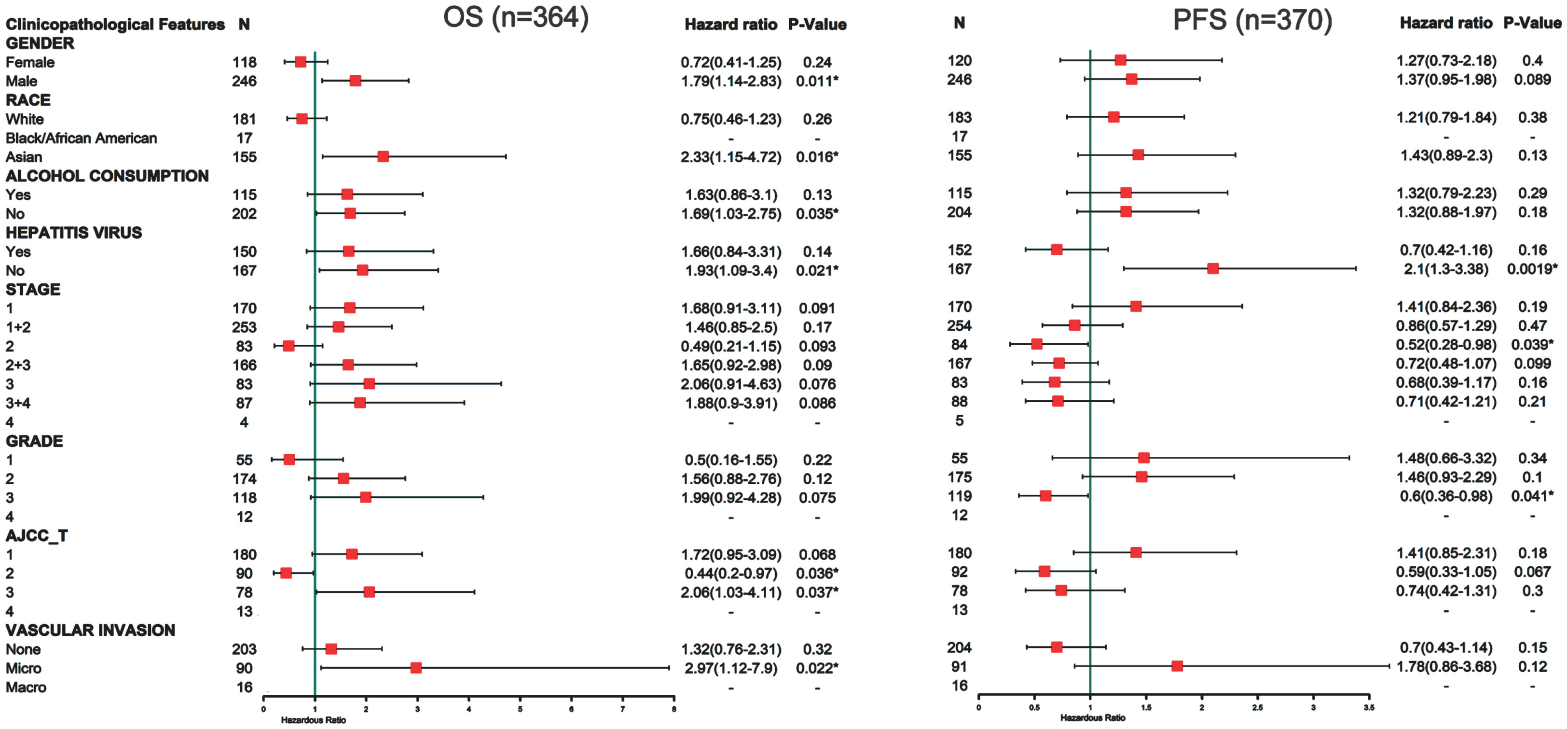
Correlation of FUNDC1 mRNA expression with OS (*n* = 364) and PFS (*n* = 370) in liver hepatocellular carcinoma with different clinicopathological features. Red squares represent hazard ratio. Short bars appear due to limited sample size for parameters and hazard ratio cannot be calculated. OS, overall survival; PFS, progression-free survival. *P < 0.05.

Next, we explored the potential relevance and underlying mechanisms of FUNDC1 expression in cancers. By integrating clinical and pathological data in Kaplan-Meier Plotter, we investigated the relationship between FUNDC1 expression and several clinical features of patients with LIHC. For OS, FUNDC1 played a detrimental role in patients with LIHC with the following characteristics: male (*n* = 246, HR = 1.79, 95% CI from 1.14 to 2.83, *P* = 0.011), Asian (*n* = 155, HR = 2.33, 95% CI from 1.15 to 4.72, *P* = 0.016), no-alcohol consumption (*n* = 202, HR = 1.69, 95% CI from 1.03 to 2.75, *P* = 0.035), no hepatitis virus infection (*n* = 167, HR = 1.93, 95% CI from 1.09 to 3.4, *P* = 0.021), AJCC T3 stage (*n* = 78, HR = 2.06, 95% CI from 1.03 to 4.11, *P* = 0.037), and micro-vascular invasion (*n* = 90, HR = 2.97, 95% CI from 1.12 to 7.9, *P* = 0.022). On the contrary, FUNDC1 expression benefited LIHC patients at AJCC T2 stage (*n* = 90, HR = 0.44, 95% CI from 0.2 to 0.97, *P* = 0.036). For progression-free survival (PFS), FUNDC1 expression was significantly hazardous to LIHC patients without hepatitis virus infection (*n* = 167, HR = 2.1, 95% CI from 1.3 to 3.38, *P* = 0.0019) but became protective to LIHC patients at stage 2 (*n* = 84, HR = 0.52, 95% CI from 0.28 to 0.98, *P* = 0.039) and grade 3 (*n* = 119, HR = 0.6, 95% CI from 0.36 to 0.98, *P* = 0.041) (Figure 4).

### Contradictory Results for LIHC and LUSC in Correlations of FUNDC1 Expression and Immune Infiltration

**Figure 5:**
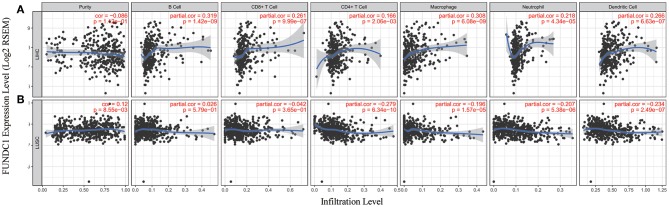
Correlation of FUNDC1 expression with immune infiltration level in LIHC and LUSC. **(A)** FUNDC1 expression has no relation with tumor purity and significant positive correlation with infiltrating levels of B cell, CD8+ T cell, CD4+ T cell, macrophage, neutrophil, and dendritic cell. **(B)** FUNDC1 expression has significant positive correlation with tumor purity, significant negative correlation with infiltrating levels of CD4+ T cell, macrophage, neutrophil and dendritic cell and no relation with infiltrating levels of B cell and CD8+ T cell. LIHC, liver hepatocellular carcinoma; LUSC, lung squamous cell carcinoma. *P* < 0.05 is considered as significant.

Immune cells in the TME can affect patient survival, and the above findings support a prognostic role of FUNDC1 in pan-cancer. Hence, it would be meaningful to explore the association between immune infiltration and FUNDC1 expression. We determined whether FUNDC1 expression was correlated with the immune infiltration level in different cancers by calculating the coefficient of FUNDC1 expression and immune infiltration level in 39 cancer types in TIMER. The results indicated that FUNDC1 expression had significant correlations with tumor purity in 11 cancer types. Furthermore, FUNDC1 expression was also significantly correlated with the infiltration levels of B cells, CD8+ T cells, CD4+ T cells, macrophages, neutrophils, and dendritic cells in 10, 20, 16, 16, 19, and 19 cancer types, respectively (Figure 5 and Supplementary Figure 3).

TIMER and GEPIA contain most of the homologous data from TCGA. Based on the above findings in GEPIA, we chose LIHC to represent cancers with poor survival and LUSC to represent cancers with good survival when FUNDC1 had a high expression level. For LIHC, the FUNDC1 expression level had significant positive correlations with the infiltration levels of B cells (*R* = 0.319, *P* = 1.42E–09), CD8+ T cells (*R* = 0.261, *P* = 9.99E–07), CD4+ T cells (*R* = 0.166, *P* = 2.06E−03), macrophages (*R* = 0.308, *P* = 6.08E–09), neutrophils (*R* = 0.218, *P* = 4.34E–05), and dendritic cells (*R* = 0.266, *P* = 6.63E−07) (Figure 5A). However, for LUSC, the FUNDC1 expression level had significant negative correlations with the infiltration levels of CD4+ T cells (*R* = −0.279, *P* = 6.34E−10), macrophages (*R* = −0.196, *P* = 1.57E−05), neutrophils (*R* = −0.207, *P* = 5.38E−06), and dendritic cells (*R* = −0.234, *P* = 2.49E−07) (Figure 5B). In addition, the FUNDC1 expression level in LIHC had no relation with tumor purity (*R* = −0.086, *P* = 0.112), while the FUNDC1 expression level in LUSC was positively correlated with tumor purity (*R* = 0.12, *P* = 8.55E-03) (Figure 5). These findings strongly suggest that FUNDC1 affects patient survival via interacting with immune infiltration in cancers like LIHC and LUSC.

### Relationships Between FUNDC1 Expression and Immune Markers

**Figure 6:**
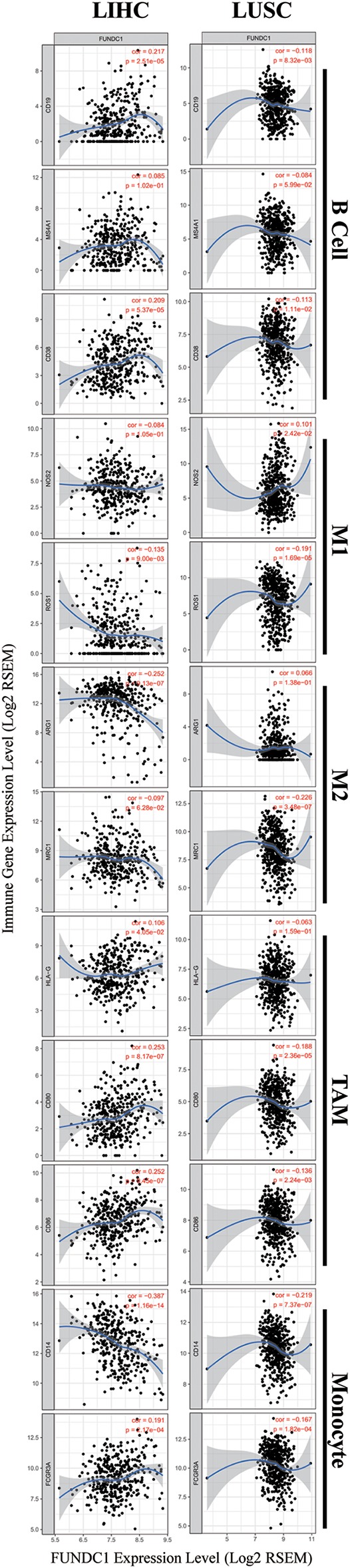
FUNDC1 expression correlates with B cell infiltration and macrophage polarization in LIHC and LUSC. Markers include CD19, MS4A1, and CD38 of B cell, NOS2 and ROS1 of M1 macrophage, ARG1 and MRC1 of M2 macrophage, HLA-G, CD80, and CD86 of TAM, and CD14 and FCGR3A of monocyte. LIHC, liver hepatocellular carcinoma; LUSC, lung squamous cell carcinoma; TAM, tumor-associated-macrophages. *P* < 0.05 is considered as significant.

To further explore the potential relationships between FUNDC1 and infiltrating immune cells, we examined the correlations between FUNDC1 and several immune cell markers in TIMER and GEPIA. These markers were used to characterize immune cells, including B cells, CD8+ T cells, M1/M2 macrophages, tumor-associated macrophages, monocytes, NK, neutrophils, and DCs in LIHC and LUSC. We also analyzed the different functional T cells such as Tfh, Th1, Th2, Th9, Th17, Th22, Treg, and exhausted T cells (Table 1 and Figure 6). In TIMER, after adjustments for tumor purity, the FUNDC1 expression level was significantly correlated with 30 out of 45 immune cell markers in LIHC and 19 out of 45 immune cell markers in LUSC (Table 1).

**Table 1:** Correlations between FUNDC1 and Gene Markers of Immune Cells in TIMER.

**Cell type**	**Gene marker**	**LIHC**				**LUSC**			
		**None**		**Purity**		**None**		**Purity**	
		**Cor**	***P***	**Cor**	***P***	**Cor**	***P***	**Cor**	***P***
B cell	CD19	0.217	^***^	0.203	^**^	−0.118	^*^	−0.058	0.203
	CD20	0.085	0.102	0.064	0.235	−0.084	0.060	−0.023	0.609
	CD38	0.209	^***^	0.197	^**^	−0.113	0.011	−0.076	0.096
CD8+ T Cell	CD8A	0.159	^*^	0.119	0.027	−0.073	0.103	−0.044	0.333
	CD8B	0.193	^**^	0.161	^*^	0.024	0.589	0.035	0.447
Tfh	CXCR5	0.178	^**^	0.163	^*^	−0.147	^**^	−0.094	0.041
	ICOS	0.199	^**^	0.171	^*^	−0.120	^*^	−0.076	0.096
	BCL-6	−0.219	^***^	−0.226	^***^	−0.006	0.888	−0.024	0.607
Th1	IL12RB2	−0.056	0.279	−0.080	0.140	0.002	0.971	0.025	0.591
	WSX-1	0.297	^***^	0.275	^***^	−0.164	^**^	−0.148	^*^
	T-BET	0.026	0.619	−0.009	0.867	−0.143	^*^	−0.105	0.022
Th2	CCR3	0.231	^***^	0.217	^***^	−0.061	0.170	−0.035	0.439
	STAT6	−0.231	^***^	−0.246	^***^	0.015	0.745	0.025	0.584
	GATA-3	0.229	^***^	0.207	^**^	−0.087	0.051	−0.060	0.191
Th9	TGFBR2	−0.194	^**^	−0.213	^***^	−0.298	^***^	−0.274	^***^
	IRF4	0.205	^***^	0.184	^**^	−0.178	^***^	−0.129	^*^
	PU.1	0.263	^***^	0.270	^***^	−0.227	^***^	−0.197	^***^
Th17	IL-21R	0.276	^***^	0.271	^***^	−0.225	^***^	−0.194	^***^
	IL-23R	0.047	0.370	0.026	0.624	−0.064	0.156	−0.033	0.468
	STAT3	0.003	0.947	−0.022	0.686	−0.181	^***^	−0.158	^**^
Th22	CCR10	0.275	^***^	0.249	^***^	−0.197	^***^	−0.167	^**^
	AHR	−0.296	^***^	−0.318	^***^	−0.160	^**^	−0.149	^*^
Treg	FOXP3	−0.014	0.787	−0.022	0.686	−0.176	^***^	−0.135	^*^
	CCR8	0.135	^*^	0.109	0.043	−0.197	^***^	−0.172	^**^
	CD25	0.197	^**^	0.183	^**^	0.024	0.559	0.081	0.077
T cell exhaustion	PD-1	0.271	^***^	0.246	^***^	−0.155	^**^	−0.116	0.011
	CTLA4	0.271	^***^	0.256	^***^	−0.126	^*^	−0.078	0.088
Macrophage	CD68	0.196	^**^	0.170	^*^	−0.202	^***^	−0.173	^**^
	CD11b	0.198	^**^	0.186	^**^	−0.232	^***^	−0.213	^***^
M1	NOS2	−0.084	0.105	−0.100	0.063	0.101	0.024	−0.087	0.057
	ROS	−0.135	^*^	−0.101	0.062	−0.191	^***^	−0.159	^**^
M2	ARG1	−0.252	^***^	−0.243	^***^	0.066	0.138	0.068	0.136
	MRC1	−0.097	0.063	−0.102	0.058	−0.226	^***^	−0.212	^***^
TAM	HLA-G	0.106	0.041	0.084	0.122	−0.063	0.159	−0.040	0.383
	CD80	0.253	^***^	0.247	^***^	−0.188	^***^	−0.161	^**^
	CD86	0.252	^***^	0.248	^***^	0.136	^*^	−0.098	0.032
Monocyte	CD14	−0.387	^***^	−0.037	^***^	−0.219	^***^	−0.190	^***^
	CD16	0.191	^**^	0.181	^**^	−0.167	^**^	−0.139	^*^
NK	XCL1	0.278	^***^	0.279	^***^	0.330	^***^	0.312	^***^
	KIR3DL1	0.003	0.951	−0.012	0.825	−0.050	0.264	−0.033	0.475
	CD7	0.239	^***^	−0.226	^***^	−0.170	0.017	−0.062	0.174
Neutrophil	CD15	0.357	^***^	0.329	^***^	−0.105	0.019	−0.060	0.194
	MPO	0.091	0.080	0.037	0.499	−0.132	^*^	−0.107	0.019
DC	CD1C	0.150	^*^	0.140	^*^	−0.078	0.081	−0.019	0.672
	CD141	−0.012	0.821	−0.059	0.277	−0.174	^***^	−0.169	^**^

*LIHC, liver hepatocellular carcinoma; LUSC, lung squamous cell carcinoma; Tfh, follicular helper T cell; Th, T helper cell; Treg, regulatory T cell; TAM, tumor-associated- macrophage; NK, natural killer cell; DC, dendritic cell; None, correlation without adjustment; Purity, correlation adjusted for tumor purity; Cor, R value of Spearman's correlation*.

* P < 0.01;

** P < 0.001;

*** *P < 0.0001*.

As shown in Figure 5, in LIHC, B cells and macrophages were two immune cell types most strongly correlated with FUNDC1 expression. However, in LUSC, these two types were less significant. According to the results in Table 1, LIHC and LUSC also have different relationships between FUNDC1 expression and B cell/macrophage markers. Therefore, we further analyzed the correlations of FUNDC1 expression and B cell/macrophage markers in tumor tissues of LIHC and LUSC in GEPIA. Notably, the results suggested that FUNDC1 correlates with tumor-associated macrophage infiltration in LIHC and LUSC (Table 2).

**Table 2:** Correlations between FUNDC1 and genes markers of B cells, macrophages, and monocytes in GEPIA.

**Cell type**	**Gene marker**	**LIHC**				**LUSC**			
		**Tumor**		**Normal**		**Tumor**		**Normal**	
		**R**	***P***	**R**	***P***	**R**	***P***	**R**	***P***
B cell	CD19	0.280	^***^	0.320	^***^	−0.220	^***^	−0.048	0.380
	CD20	0.130	0.015	0.160	0.041	−0.180	^***^	−0.190	^**^
	CD38	0.300	^***^	0.220	^**^	−0.210	^***^	0.290	^***^
M1	NOS2	0.110	0.042	0.260	^**^	0.170	^**^	−0.300	^***^
	ROS	0.060	0.250	0.110	0.160	−0.130	^*^	0.360	^***^
M2	ARG1	−0.270	^***^	0.380	^***^	0.073	0.110	−0.160	^*^
	MRC1	0.039	0.450	0.350	^***^	−0.170	^**^	0.320	^***^
TAM	HLA–G	0.230	^***^	0.350	^***^	−0.150	^**^	0.130	0.021
	CD80	0.400	^***^	0.330	^***^	−0.210	^***^	−0.240	^***^
	CD86	0.390	^***^	0.450	^***^	−0.180	^***^	0.016	0.770
Monocyte	CD14	−0.410	^***^	0.190	0.015	−0.230	^***^	0.140	^*^
	CD16	0.340	^***^	0.480	^***^	−0.180	^***^	0.320	^***^

*LIHC, liver hepatocellular carcinoma; LUSC, lung squamous cell carcinoma; TAM, tumor-associated-macrophage; Tumor, correlation analysis in tumor tissue of TCGA; Normal, correlation analysis in normal tissue of TCGA*.

* P < 0.01;

** P < 0.001;

*** *P < 0.0001*.

Moreover, FUNDC1 expression in LIHC and LUSC also relates differently with CD8+ T cell, Tfh, Th2, Th17, and Treg infiltration. The relationships of FUNDC1 with Th9 cells, Th22 cells, neutrophils, and NK cells were partially different between LIHC and LUSC. Additionally, FUNDC1 in LIHC also had significant correlations with exhausted T cell markers such as PD-1 and CTLA4, while FUNDC1 in LUSC did not have such a link (Table 1). Hence, these results confirm our speculation that FUNDC1 expression in LIHC and LUSC correlates with immune cell infiltration in different manners, which can help explain the differences in patient survival.

## Discussion

Mitochondria are essential for human immunity, and aberrant mitochondrial activity affects immune responses. For example, some studies have demonstrated that viruses (e.g., HBV in LIHC) can manipulate mitophagy, which enables viruses to promote persistent infection and attenuate the innate immune responses (25). FUNDC1 is an integral mitochondrial outer-membrane protein and a receptor for hypoxia-induced mitophagy. Unlike ubiquitin-dependent mitophagy, which is mainly mediated by PINK1 (PTEN induced kinase-1)-PRKN/PARK2 (parkin RBR E3 ubiquitin protein ligase), FUNDC1 is an LC3 interacting region-containing receptors that can directly induce mitophagy (26). FUNDC1-mediated mitophagy is negatively regulated by the phosphorylation of FUNDC1, as the phosphorylation of Tyr 18 in the FUNDC1 LC3-interacting region motif can weaken the binding affinity of FUNDC1 to LC3 (5). Meanwhile, it is now clear that hypoxia, a common feature of cancers, can induce extensive mitochondrial degradation in a FUNDC1-dependent manner (27). Hypoxia can induce FUNDC1 dephosphorylation and therefore enhance its selective interaction with LC3 (4, 5). In contrast, hypoxia can also promote the ubiquitylation of FUNDC1 at lysine 119 and subsequent degradation of redundant FUNDC1 through MARCH5, a mitochondrial ubiquitin ligase that fine-tunes hypoxia-induced mitophagy. However, severe hypoxic stress still leads to the dephosphorylation of FUNDC1 and increased mitophagic flux (28).

Although FUNDC1 has not been well-studied in immuno-oncology, several studies have been conducted. It is now acknowledged that mitophagy is closely related to human immunity. Moreover, to the best of our knowledge, the relationship between FUNDC1 expression and tumor cell proliferation has been proved in cervical and breast cancers (6, 29). Thus, it is reasonable to surmise that FUNDC1 expression may influence patient survival through tumor cell proliferation. However, the role of FUNDC1 in other important aspects like tumor metastasis has not been thoroughly studied. As indicated by previous reports and our findings, it should also be noted that the role of FUNDC1 may vary in different contexts. Although this study offers a broad view related to patient survival, additional downstream mechanism studies are still warranted. One previous study has demonstrated that FUNDC1-mediated mitophagy can suppress hepatocarcinogenesis (11). This study found that the specific knockout of FUNDC1 in hepatocytes promotes the initiation and progression of chemical carcinogen diethylnitrosamine-induced HCC. The study also found that FUNDC1 transgenic hepatocytes protect against the development of HCC. However, according to our study, FUNDC1 is an unfavorable prognostic factor for LIHC patients (Figure 3), though the situation may vary according to different characteristics such as gender, race, alcohol consumption, hepatitis infection, tumor grade, tumor stage, or vascular invasion (Figure 4). The differences between our study using a cancer patient cohort and previous study using an animal model necessitate more comprehensive and precise studies in the future to explain tumor development. Understanding the tumor microenvironment, including immune cell infiltration, can probably help decipher the mechanisms behind tumor development. As shown in Figures 5, 6, we did find significant correlations between tumor FUNDC1 expression and immune cell infiltration, even though a cause-effect relationship could not be established in the current study. Besides, FUNDC1 expression is not related with the tumor purity in LIHC, while it does have significant positive correlation with the tumor purity in LUSC (Figure 5). Such difference may be attributable to the different enrichment patterns of FUNDC1 in the tumor microenvironment. Tumor microenvironment is a complex milieu of non-cancerous cells mainly consisting of immune cells around tumor cells. Genes highly expressed in cells in the microenvironment are believed to have negative associations with tumor purity. In contrast, genes highly expressed in the tumor cells are expected to have positive association with tumor purity. In this study, as an essential regulator of mitophagy, FUNDC1 expression have different relationships with tumor purity in different contexts, which suggests FUNDC1-related differences in various aspects such as carcinogenesis, metastasis, treatment strategies, etc. Future studies using more precise techniques such as single cell RNA sequencing and exploring direct interactions at the cellular and molecular levels are needed.

In this study, we demonstrated the prognostic value of FUNDC1 in pan-cancer. Compared with a low expression level, a high expression level of FUNDC1 correlated with a better prognosis in lung, ovarian, kidney, and thyroid cancers and a poorer prognosis in brain, skin, and liver cancers. Interestingly, increased FUNDC1 expression can specifically impact the prognosis of LIHC patients with the following characteristics: male, Asian, no alcohol consumption, no hepatitis virus infection, stage 2, grade 3, AJCC T2 and T3, and micro-vascular invasion. Our analysis also revealed that LIHC and LUSC have different patterns with respect to correlations between immune infiltration and FUNDC1 expression. Hence, this study provides insights into the use of the mitophagy regulator FUNDC1 as a prognostic marker in pan-cancer from an immuno-oncological perspective, which could benefit future mechanistic studies and aid in the development of immunotherapies.

This study explored the expression levels of FUNDC1 and visualized the prognostic landscape in pan-cancer using independent datasets in ONCOMINE and PROGNOSCAN and TCGA data in GEPIA and TIMER. In ONCOMINE, we found that FUNDC1, compared with expression levels in normal tissues, was highly expressed in breast, cervical, colorectal, lung, ovarian, pancreatic, and prostate cancers, as well as in leukemia and lymphoma, while only one dataset showed that FUNDC1 had a lower expression level in breast cancer (Figure 1A). However, analysis of TCGA data in TIMER revealed that FUNDC1 expression was higher in BLCA, BRCA, CHOL, COAD, LIHC, LUAD, LUSC, PRAD, and UCEC and lower in KIRC compared with that in normal adjacent tissues (Figure 1B). These discrepant FUNDC1 expression levels in cancers across different databases are the result of heterogeneous data collection approaches, as well as underlying mechanisms with distinct biological properties. However, across databases, we identified the consistent prognostic value of FUNDC1 expression in LIHC and LUSC. For patient prognosis, analysis of FUNDC1 in Kaplan-Meier Plotter and GEPIA revealed that increased FUNDC1 expression correlated with a favorable prognosis in LUSC as well as an overall beneficial effect on cancers. In contrast, a high expression level of FUNDC1 in LIHC was correlated with a poor prognosis. In 8 datasets in PrognoScan, high FUNDC1 expression levels could be used as an independent risk factor for a poor prognosis in brain, breast, colorectal, and skin cancers (Figure 2). In addition, a high level of FUNDC1 expression was shown to be related to a poor prognosis in LIHC with micro-vascular invasion but a favorable prognosis in LIHC with AJCC T2 (Figure 4). Taken together, these findings strongly suggest that FUNDC1 can serve as a prognostic biomarker in pan-cancer.

Another major finding from this study is that FUNDC1 expression correlates with diverse immune infiltration levels in cancers, especially in LIHC and LUSC. Our findings demonstrate that FUNDC1 expression has significant relationships with the infiltration level of CD4+ T cells, macrophages, neutrophils, and DCs in LIHC and LUSC (Figure 5). LIHC has positive coefficients between immune infiltration and the FUNDC1 expression level, but LUSC generally has negative ones. As we expected, the relationships between FUNDC1 expression and certain immune cell markers, such as NOS2, ROS1, ARG1, MRC1, and CD14, are not always the same as the overall trend, suggesting that specific interactions between FUNDC1 and certain immune cell subtypes (Figure 5 and Table 1). Interestingly, the FUNDC1 expression level in LIHC is not related to tumor purity, suggesting that it is equally expressed in tumor cells and the tumor microenvironment. However, in LUSC, the FUNDC1 expression level had a significant positive correlation with tumor purity, indicating its comparative enrichment in tumor cells. As important antigen presenting cells, B cells and macrophages were the cell types that most significantly correlated with FUNDC1 expression in LIHC; however, in LUSC, B cells had no relation to FUNDC1, and macrophages had the lowest coefficient with FUNDC1 among all significantly correlated cells (Figure 5). These differences suggest heterogeneities among tumors in recruiting antigen presenting cells to the TME. Moreover, after adjustment for tumor purity, FUNDC1 in LIHC had no relation to M1 macrophages but negatively correlated with ARG1+ M2 macrophages, while FUNDC1 in LUSC negatively correlated with ROS+ M1 macrophages and MRC1+ M2 macrophages. For infiltrating TAMs, after adjustment for tumor purity, in LIHC, FUNDC1 was positively correlated with CD80 and CD86, and in LUSC, FUNDC1 negatively correlated with CD80, indicating the constitutive effect of TAMs on differences in tumor cell survival (Table 1). Taken together, these findings suggest that FUNDC1 plays an important role in the recruitment and regulation of immune infiltrating cells in cancers, which may eventually influence patient survival.

Recent studies provide possible mechanisms explaining why FUNDC1 expression correlates with immune infiltration and different prognoses in pan-cancer. Initial theories assumed that immune cells helped resisting tumors. Indeed, it is still acknowledged that in the early phase of carcinogenesis, the immune system attacks tumor cells by activating T cells and macrophages to prevent the development of cancer. However, once a tumor progress past this early stage, the immune TME switches to supporting cancer cells and promoting tumor progression while suppressing immune cell-mediated cytotoxicity (12). FUNDC1 is a key regulator of mitophagy, which has been proved to participate in immunity (26). Several studies have linked mitophagy to mitochondrial antigen presentation (26, 30). Our study also provided evidence that the immune infiltration levels of antigen presenting cells (B cells and macrophages) are significantly correlated with the FUNDC1 expression level in cancers. The role of mitophagy in antigen presentation remains unclear. While one study reported that mitophagy can reduce CD8+ T cell activation, another study showed its facilitating role in the mitochondrial antigen presentation of glycoproteins. Aside from antigen presentation, mitophagy is also involved in the development and differentiation of immune cells, including T cells, natural killer (NK) cells, and macrophages (26).

Apart from interactions among immune cells, tumor cells can influence immune cells by producing lactic acid (31). This signal can induce the M2-like polarization of tumor-associated macrophages. In turn, the lactate-induced expression of arginase 1 by macrophages also plays an important role in tumor growth. Macrophages can also promote carcinogenesis by producing pro-inflammatory mediators such as IL-6, tumor necrosis factor, interferon-γ, proteases, ROS, and nitrogen species (32, 33). Specifically, TAMs can directly help tumor cell migration via a paracrine loop between macrophages and tumor cells that involves the secretion of EGF family ligands from macrophages and CSF1 from tumor cells. TAMs can also produce cathepsins and matrix-remodeling enzymes to stimulate such process and increase tumor invasiveness (34–36). These interactions between tumor cells and infiltrating immune cells help explain the findings from this study indicating that TAMs have a positive correlation with FUNDC1 expression in LIHC and that the high expression of FUNDC1 is associated with a worse LIHC patient prognosis. For other immune cell types, T helper 2 (TH2) cells in the microenvironment can educate macrophages to become pro-tumoral and alter the immune response from a cytotoxic to a supportive role (15). Tumor-associated neutrophil and plasma cell signatures can also serve as significant but opposite predictors of survival for diverse solid tumors (37). Therefore, it is reasonable to surmise that immune infiltration can interact with FUNDC1-mediated activities in both immune cells and tumor cells.

However, even though we integrated information across multiple databases, this study still had limitations. First, a large proportion of the microarray and sequencing data were collected by analyzing tumor tissue information. Thus, the cell-level analysis of immune cell markers could have introduced systematic bias. To overcome this issue, future studies with a higher resolution, such as with the use of single cell RNA sequencing, should be performed (38, 39). Second, there is no information in these databases reflecting the post-translational modification of FUNDC1. As discussed above, both phosphorylation and ubiquitination can interfere with the molecular function of FUNDC1. Third, we still cannot define FUNDC1 as a friend or foe because of some conflicting findings from different databases. Fourth, this study only conducted a bioinformatics analysis of FUNDC1 expression and patient survival across different databases, and *in vivo*/*in vitro* experiments were not performed. Future mechanistic studies on FUNDC1 at the cellular and molecular levels could help clarify the role of FUNDC1. Fifth, despite the finding that FUNDC1 expression correlates with both immune cell infiltration and patient survival in cancers, we could not prove that FUNDC1 affects patient survival through immune infiltration. Future prospective studies focusing on FUNDC1 expression and immune infiltration in a cancer population could help provide a definitive answer.

In summary, FUNDC1 can affect pan-cancer prognosis and correlate with immune infiltration. Specially, for LIHC and LUSC, FUNDC1 is related to different TAM patterns in the TME. FUNDC1 can serve as a prognostic biomarker in pan-cancer. These findings may provide an immuno-based anti-tumor strategy involving manipulating the energy system of either tumor cells or tumor microenvironment infiltrates.

## Data Availability Statement

Publicly available datasets were analyzed in this study. This data can be found here: www.oncomine.org, http://dna00.bio.kyutech.ac.jp/PrognoScan/index.html, https://kmplot.com/analysis/, http://gepia.cancer-pku.cn, http://cistrome.org/TIMER/.

## Author Contributions

QY, NS, and XC designed this study. NS, JZ, YW, XY, and WM extracted the information from the databases. QY and NS analyzed the data. YL and XC supervised the entire study. QY wrote the manuscript. All authors revised the manuscript.

### Conflict of Interest

The authors declare that the research was conducted in the absence of any commercial or financial relationships that could be construed as a potential conflict of interest.

## Abbreviations

BLCA: bladder urothelial carcinoma
BRCA: breast invasive carcinoma
CHOL: cholangiocarcinoma
COAD: colon adenocarcinoma
DFS: disease-free survival
DMFS: distant metastasis-free survival
DSS: disease-specific survival
FUNDC1: FUN14 domain containing 1
HNSC: head and neck squamous cell carcinoma
HNSC-HPVpos: head and neck squamous carcinoma-HPV positive
HR: hazard ratio
KICH: kidney chromophobe
KIRC: kidney renal clear cell carcinoma
LGG: brain lower grade glioma
LIHC: liver hepatocellular carcinoma
LUAD: lung adenocarcinoma
LUSC: lung squamous cell carcinoma
OS: overall survival
OV: ovarian cancer
PFS: progression-free survival
PRAD: prostate adenocarcinoma
READ: rectum adenocarcinoma
RFS: relapse-free survival
TAM: tumor-associated-macrophage
THCA: thyroid carcinoma
TME: tumor microenvironment
UCEC: uterine corpus endometrial carcinoma.
