# Supplementary material for: Prognostic and Immunological Role of FUN14 Domain Containing 1 in Pan-Cancer: Friend or Foe?
Source: Front Oncol. 2020 Jan 10;9:1502. doi: 10.3389/fonc.2019.01502 (PMC6966411; doi:10.3389/fonc.2019.01502)
Supplement: Supplementary file 1 [file Table_1.docx]

**Supplementary Table**

Supplementary Table 1. FUNDC1 expression in cancerous versus normal tissue in ONCOMINE.

| **Cancer Site** | **Cancer Type** | **P Value** | **Sample Size** | **Fold Change** | **Up/Down Regulated  Gene Rank** | **Reference  (PMID)** |
| --- | --- | --- | --- | --- | --- | --- |
| Breast | Ductal Breast Carcinoma in Situ Epithelia | 2.08E-05 | 9 | 3.119 | 166(1%) | 19187537 |
|  | Invasive Ductal Breast Carcinoma Epithelia | 7.58E-05 | 9 | 2.600 | 232(2%) | 19187537 |
|  | Tubular Breast Carcinoma | 2.40E-27 | 67 | 1.938 | 272(2%) | 22522925 |
|  | Breast Carcinoma | 3.68E-06 | 14 | 1.657 | 327(2%) | 22522925 |
|  | Invasive Ductal and Invasive Lobular Breast Carcinoma | 5.78E-28 | 90 | 1.911 | 406(3%) | 22522925 |
|  | Invasive Breast Carcinoma | 1.48E-06 | 21 | 1.778 | 553(3%) | 22522925 |
|  | Mucinous Breast Carcinoma | 1.82E-14 | 46 | 1.677 | 678(4%) | 22522925 |
|  | Invasive Lobular Breast Carcinoma | 1.71E-29 | 148 | 1.600 | 849(5%) | 22522925 |
|  | Invasive Ductal Breast Carcinoma | 8.19E-59 | 1556 | 1.609 | 1073(6%) | 22522925 |
|  | Invasive Ductal and Lobular Carcinoma | 9.70E-04 | 3 | 1.538 | 1274(7%) | TCGA |
|  | Male Breast Carcinoma | 1.11E-04 | 9 | -1.670 | 414(3%) | TCGA |
| Cervical | Cervical Cancer | 4.48E-08 | 20 | 2.803 | 830(5%) | 17510386 |
| Colorectal | Rectosigmoid Adenocarcinoma | 1.17E-04 | 10 | 1.922 | 1388(8%) | 17615082 |
|  | Rectal Adenocarcinoma | 4.71E-04 | 8 | 1.558 | 1427(8%) | 17615082 |
|  | Colon Adenoma | 6.46E-05 | 5 | 1.547 | 1669(9%) | 20957034 |
|  | Colon Adenoma | 5.31E-09 | 25 | 1.624 | 1952(10%) | 18171984 |
| Leukemia | B-Cell Acute lymphoblastic Leukemia | 1.84E-22 | 147 | 1.600 | 1267(8%) | 19486012 |
| Lung | Lung Adenocarcinoma | 1.35E-15 | 226 | 1.727 | 293(2%) | 22080568 |
|  | Lung Adenocarcinoma | 5.70E-12 | 45 | 1.607 | 433(3%) | 20421987 |
|  | Squamous Cell Lung Carcinoma | 8.73E-12 | 27 | 1.761 | 575(3%) | 20421987 |
| Lymphoma | Angioimmunoblastic T-Cell Lymphoma | 7.38E-05 | 6 | 2.251 | 1888(10%) | 17304354 |
| Ovarian | Ovarian Serous Adenocarcinoma | 5.93E-05 | 43 | 1.592 | 1267(8%) | 19486012 |
| Pancreatic | Pancreatic Carcinoma | 1.93E-06 | 36 | 1.724 | 636(4%) | 19732725 |
|  | Pancreatic Ductal Adenocarcinoma | 5.93E-08 | 39 | 1.729 | 1851(10%) | 19260470 |
| Prostate | Prostate Adenocarcinoma | 5.26E-04 | 27 | 1.923 | 1224(7%) | 12873976 |
