# Supplementary figures and images for: Prognostic and Immunological Role of FUN14 Domain Containing 1 in Pan-Cancer: Friend or Foe?
Source: Front Oncol. 2020 Jan 10;9:1502. doi: 10.3389/fonc.2019.01502 (PMC6966411; doi:10.3389/fonc.2019.01502)

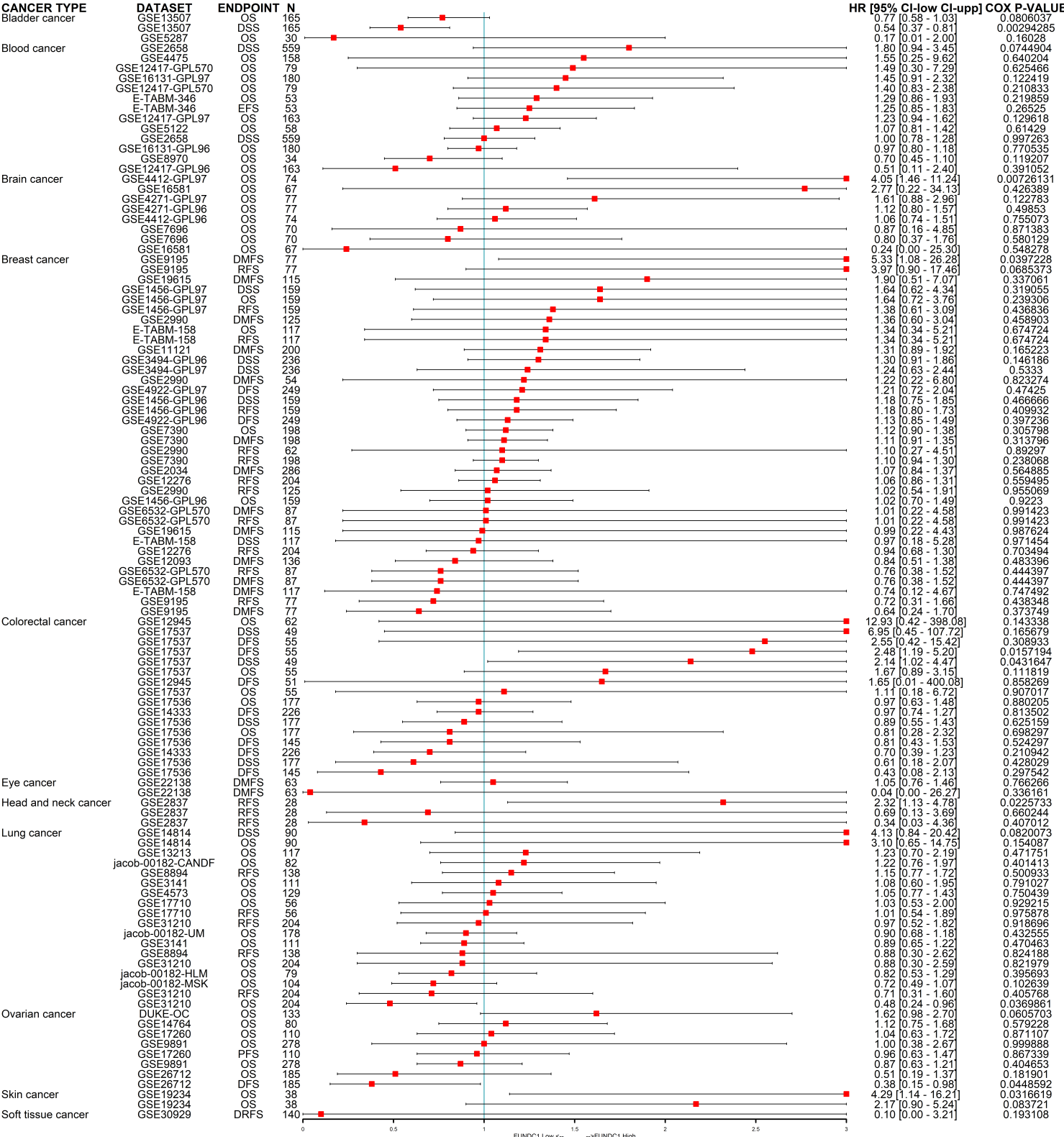

Supplement: Supplementary file 2 [file Data_Sheet_1.PDF]

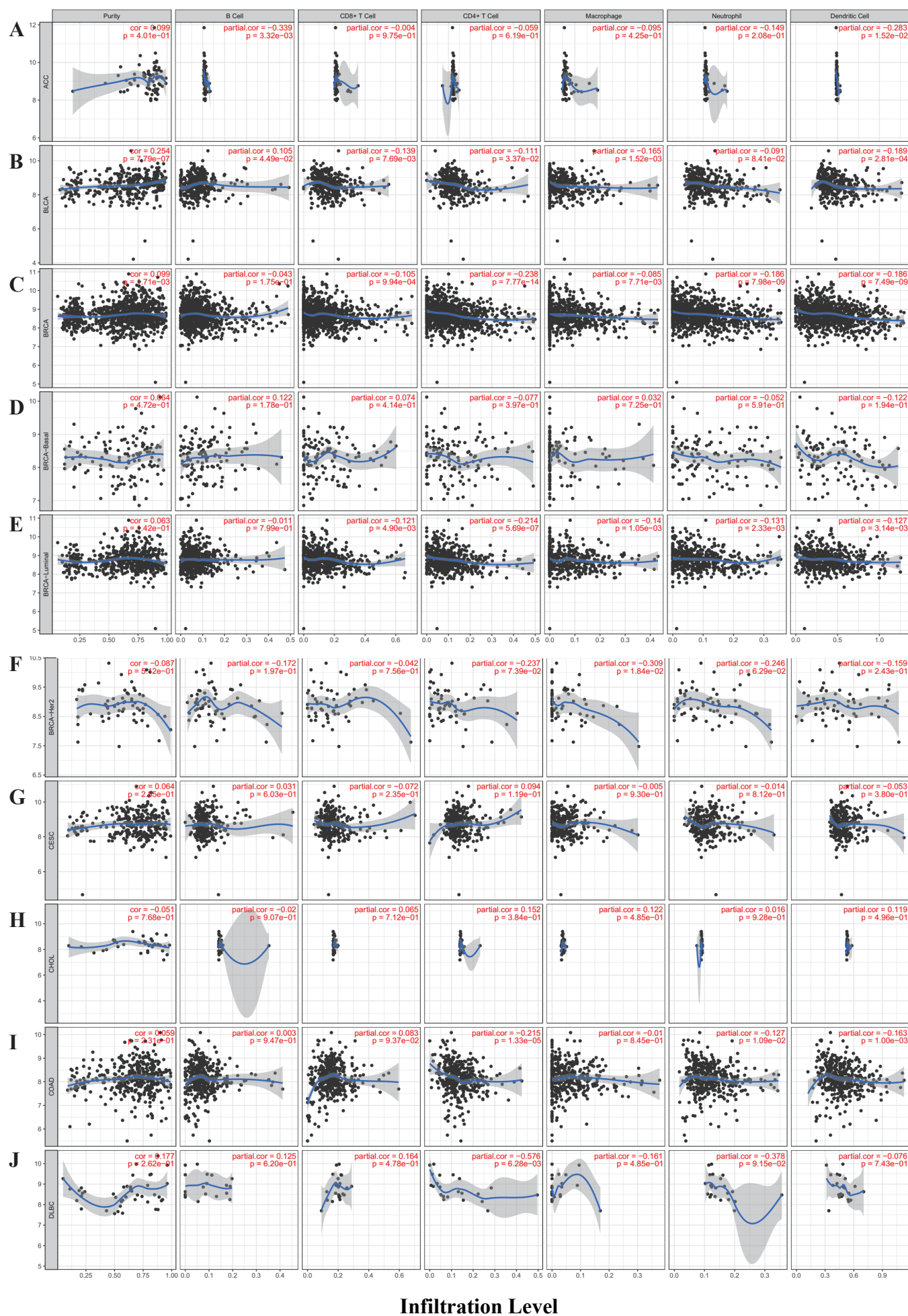

# FUNDC1 Expression Level (Log2 RSEM)

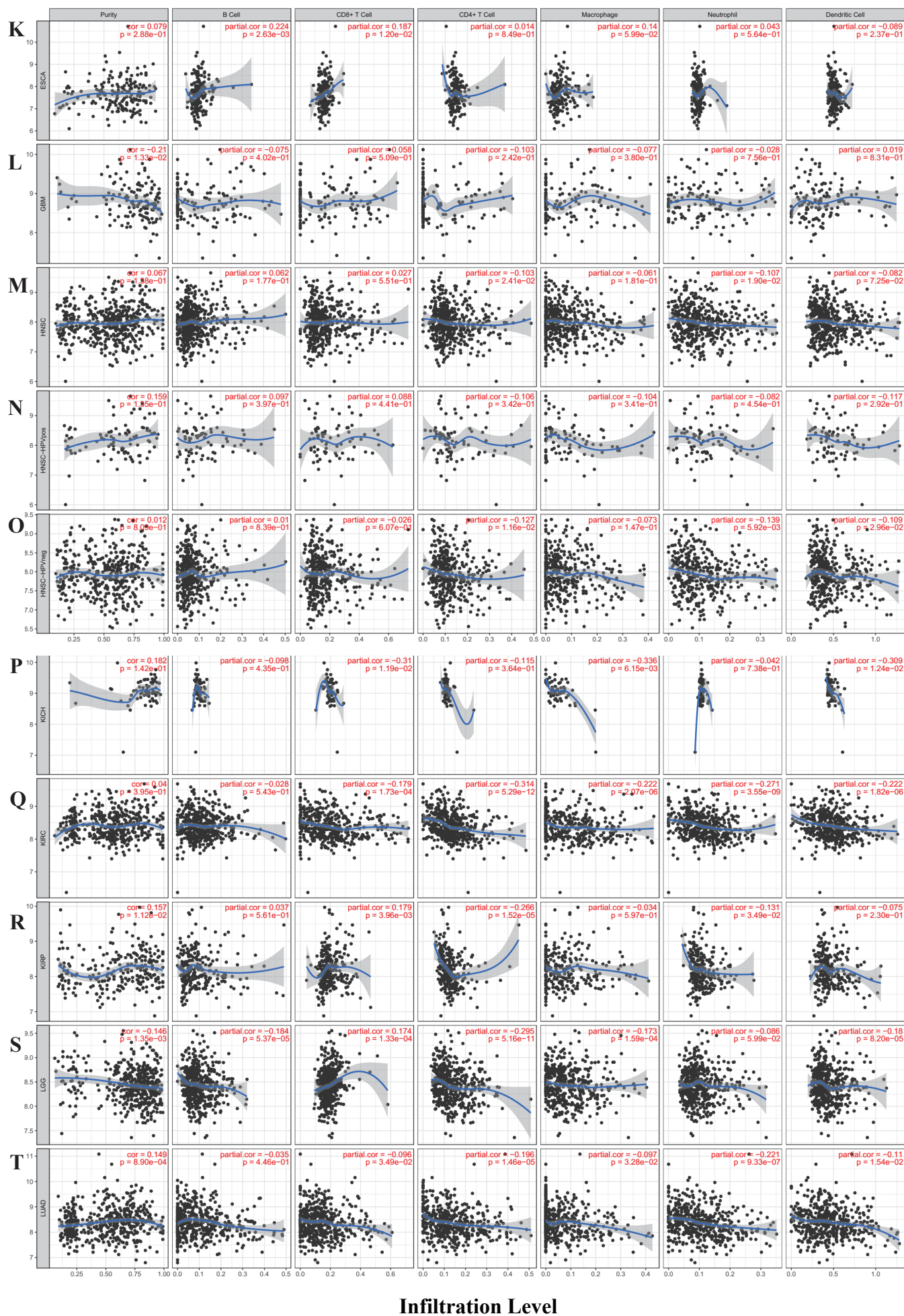

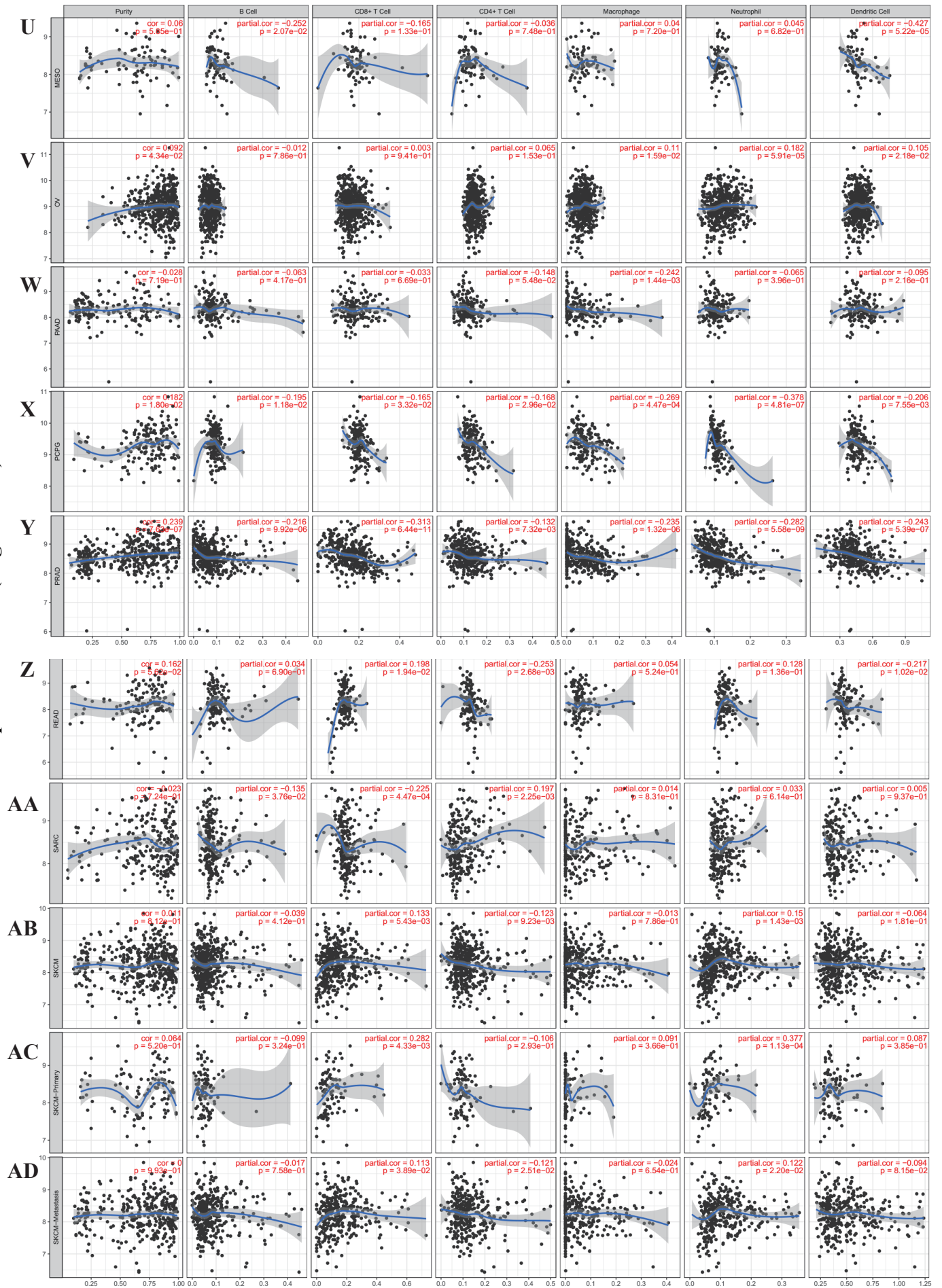

FUNDCl Expression Level (Log2 RSEM)

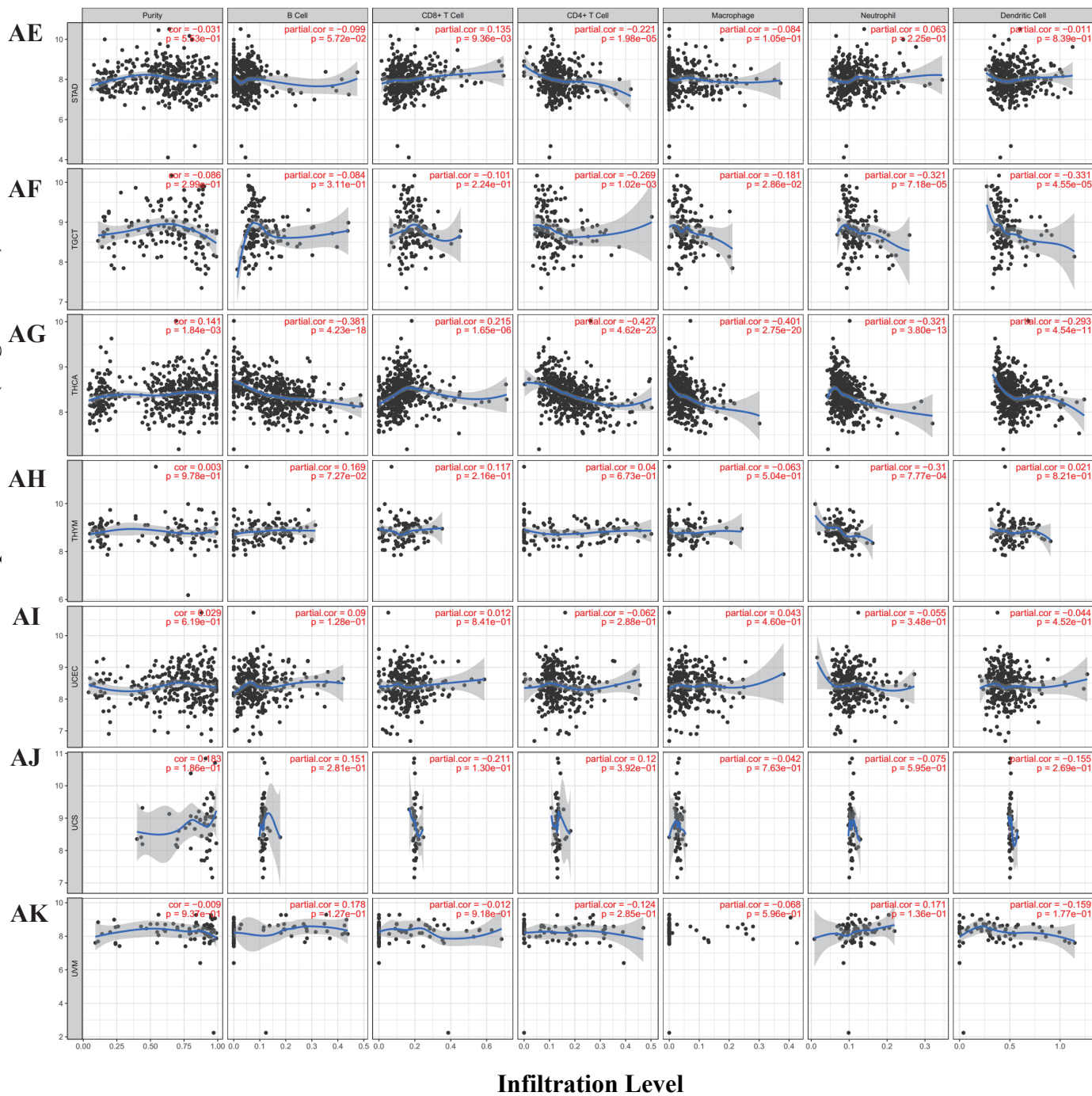

Supplement: Supplementary file 3 [file Data_Sheet_2.PDF]

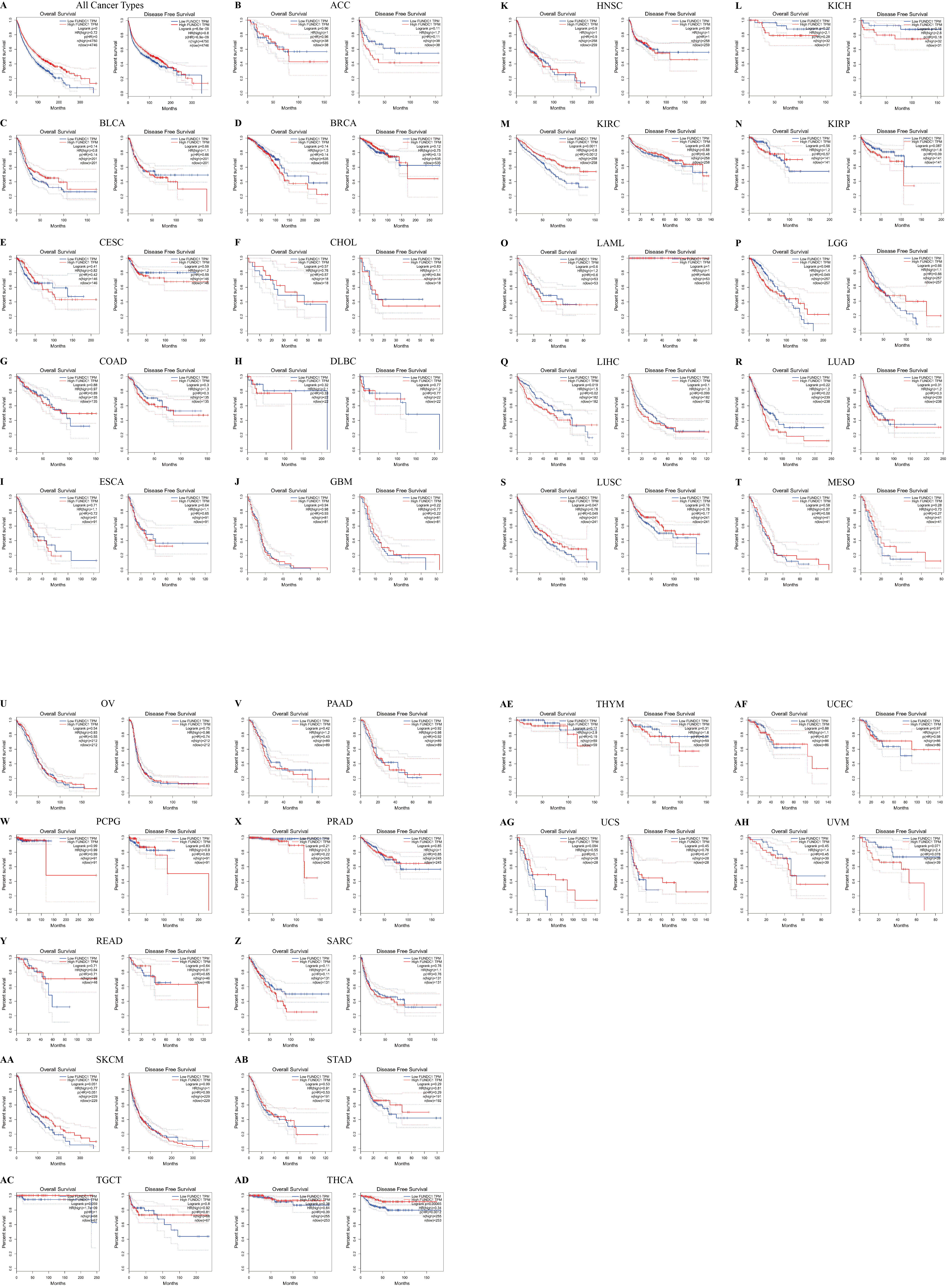

Supplement: Supplementary file 5 [file Image_1.JPEG]
