# Supplementary material for: Prognostic and Immunological Role of FUN14 Domain Containing 1 in Pan-Cancer: Friend or Foe?
Source: Front Oncol. 2020 Jan 10;9:1502. doi: 10.3389/fonc.2019.01502 (PMC6966411; doi:10.3389/fonc.2019.01502)
Supplement: Supplementary file 4 [file Data_Sheet_3.docx]

**Supplementary Figure Legends**

**Supplementary Figure 1.** Relation between FUNDC1 expression and patient prognosis of different datasets of cancers in PrognoScan. Red square represents hazard ratio. OS, overall survival; DSS, disease-specific survival; EFS, event-free survival; DMFS, distant metastasis-free survival; RFS, relapse-free survival; DFS, disease-free survival.

**Supplementary Figure 2.** Correlation of FUNDC1 expression with prognostic values in cancers. Overall survival and disease free survival of **(A)** all cancer types **(B)** ACC, Adrenocortical carcinoma **(C)** BLCA, Bladder Urothelial Carcinoma **(D)** BRCA, Breast invasive carcinoma **(E)** CESC, Cervical squamous cell carcinoma and endocervical adenocarcinoma **(F)** CHOL, Cholangio carcinoma **(G)** COAD, Colon adenocarcinoma **(H)** DLBC, Lymphoid Neoplasm Diffuse Large B-cell Lymphoma **(I)** ESCA, Esophageal carcinoma **(J)** GBM, Glioblastoma multiforme **(K)** HNSC, Head and Neck squamous cell carcinoma **(L)** KICH, Kidney Chromophobe **(M)** KIRC, Kidney renal clear cell carcinoma **(N)** KIRP, Kidney renal papillary cell carcinoma **(O)** LAML, Acute Myeloid Leukemia **(P)** LGG, Brain Lower Grade Glioma **(Q)** LIHC, Liver hepatocellular carcinoma **(R)** LUAD, Lung adenocarcinoma **(S)** LUSC, Lung squamous cell carcinoma **(T)** MESO, Mesothelioma **(U)** OV, Ovarian serous cystadenocarcinoma **(V)** PAAD, Pancreatic adenocarcinoma **(W)** PCPG, Pheochromocytoma and Paraganglioma **(X)** PRAD, Prostate adenocarcinoma **(Y)** READ, Rectum adenocarcinoma **(Z)** SARC, Sarcoma **(AA)** SKCM, Skin Cutaneous Melanoma **(AB)** STAD, Stomach adenocarcinoma **(AC)** TGCT, Testicular Germ Cell Tumors **(AD)** THCA, Thyroid carcinoma **(AE)** THYM, Thymoma **(AF)** UCEC, Uterine Corpus Endometrial Carcinoma **(AG)** UCS, Uterine Carcinosarcoma and **(AH)** UVM, Uveal Melanoma.

**Supplementary Figure 3.** Correlation of FUNDC1 expression with immune infiltration levels in cancers via TIMER database. Correlation of FUNDC1 expression with immune infiltration levels in **(A)** ACC, Adrenocortical carcinoma **(B)** BLCA, Bladder Urothelial Carcinoma **(C)** BRCA, Breast invasive carcinoma **(D)** BRCA-Basal, Breast invasive carcinoma-Basal **(E)** BRCA-Luminal, Breast invasive carcinoma-Luminal **(F)** BRCA-Her2, Breast invasive carcinoma-Her2 **(G)** CESC, Cervical squamous cell carcinoma and endocervical adenocarcinoma **(H)** CHOL, Cholangio carcinoma **(I)** COAD, Colon adenocarcinoma **(J)** DLBC, Lymphoid Neoplasm Diffuse Large B-cell Lymphoma **(K)** ESCA, Esophageal carcinoma **(L)** GBM, Glioblastoma multiforme **(M)** HNSC, Head and Neck squamous cell carcinoma **(N)** HNSC-HPVpos, Head and Neck squamous cell carcinomaHPVpos **(O)** HNSC-HPVneg, Head and Neck squamous cell carcinomaHPVneg **(P)** KICH, Kidney Chromophobe **(Q)** KIRC, Kidney renal clear cell carcinoma **(R)** KIRP, Kidney renal papillary cell carcinoma **(S)** LGG, Brain Lower Grade Glioma **(T)** LUAD, Lung adenocarcinoma **(U)** MESO, Mesothelioma **(V)** OV, Ovarian serous cystadenocarcinoma **(W)** PAAD, Pancreatic adenocarcinoma **(X)** PCPG, Pheochromocytoma and Paraganglioma **(Y)** PRAD, Prostate adenocarcinoma **(Z)** READ, Rectum adenocarcinoma **(AA)** SARC, Sarcoma **(AB)** SKCM, Skin Cutaneous Melanoma **(AC)** SKCM-Primary, Skin Cutaneous Melanoma-Primary **(AD)** SKCM-Metastasis, Skin Cutaneous Melanoma-Metastasis **(AE)** STAD, Stomach adenocarcinoma **(AF)** TGCT, Testicular Germ Cell Tumors **(AG)** THCA, Thyroid carcinoma **(AH)** THYM, Thymoma **(AI)** UCEC, Uterine Corpus Endometrial Carcinoma **(AJ)** UCS, Uterine Carcinosarcoma and **(AK)** UVM, Uveal Melanoma.
